# Gelseminum Sempervirens

**Published:** 1877-03-20

**Authors:** 


### GELSEMINUM SEMPERVIRENS.

The depressing influence of the *gelseminum sempervirens* upon the circulation has been known for many years, and Southern practitioners have employed it in the treatment of fever for over a quarter of a century. The joint experimental observations of Ringer and Murrell (*Lancet*, 1876,) recently made, whilst confirmatory of those made by Bartholow and Ott, have added further information concerning the effects of this valuable and interesting remedy. To test its action on man, it was given in doses to occasion its toxic effects. The tincture (one part of root to four of rectified spirits) was given in drachm doses, hourly for three hours without endangering the life of the subject. The symptoms generally occur in a certain order. The drug produces *pain* in the eyes and brow, followed by giddiness, (which, standing or walking, measurably increases) and soon after by dimness of sight; diplopia, with contracted pupils; dropping of the eye-lids, with restricted movements of the eye-balls. The last complaint (beyond which the remedy was not pushed) is weakness of the legs.

When fully under its influence, the patient is pale, with a sleepy look—heavy eyes and a disposition to yawn frequently. There is a sense of dryness of the mouth, though the tongue looks and feels to the finger moist. The experimental investigations of Ringer and Murrell, also those by Dr. J. Burden Sanderson (*Lancet*, April 1, 1876), clearly show that gelseminum exerts its remarkable control over the respiration by its direct influence upon the automatic respiratory center.

The effects occasioned by the local application of gelseminum, and the internal administration, were, singular to say, of an opposite character. In doses sufficiently large to occasion its characteristic symptoms, *contraction* of the pupil ensued (Ringer and Murrell). The local use of the alkaloid *dilates* the pupil.

The physiological antagonism between gelseminum and strychnia is clearly demonstrated by the experiments upon frogs by Ringer and Murrell. The strychnia convulsions were suppressed in from twenty-five minutes to an hour and a half, by the use of the gelseminum; whereas, without a resort to it the convulsions lasted two or three days. The experiments serve as sign-boards to the therapeutical usefulness to be anticipated from the employment of the remedy in all conditions indicative of an exaltation of the spinal functions. Indeed, several cases of tetanus have been recorded as fully relieved by this remedy.

The writer has repeatedly verified in his experiences the published statements of Drs. Legg, Spencer Thompson, Sawyer (*Brit. Med. Jour*, May, 1874,) as to the efficacy of gelseminum in facial neuralgia, especially in that phase of the affection involving the teeth and alveolar processes of the jaw. The writer, for some years past, has employed this remedy in acute inflammatory disorders of the respiratory tract. And his clinical experience attests the therapeutical value of this remedy as a reliable and somewhat speedy repressive of the febrile commotion consequent upon the local disturbances. In the treatment of obstinate cases of intermittent fever, the writer is satisfied, from observation of the superior efficacy of a combination of quinine and gelseminum.

During the last two years the writer has had frequent occasion to test the influence of this remedy in cases of rigid os uteri and sphincter perinei. The plan adopted was the administration of fifteen drops of the fluid extract of gelseminum, at an interval of a quarter of an hour, until some decided results ensued. It affords him pleasure to be able to affirm the potency of this agent in overcoming the sphincteric barriers to delivery, especially as we owe the suggestion to a distinguished Honorary Fellow of this Society, Dr. R. S. Payne, of Lynchburg (*Virginia Med. Monthly*, December, 1874). The following interesting and unique case is briefly submitted in further illustration of the therapeutic uses of gelseminum. The writer was called in consultation by Dr. William H. Byerly, of Rockingham county. The following history was elicited: In the *three* previous confinements, from the irregular contractions—partially affecting the muscular fibres—without uniform hardening of the uterine globe; from the exhaustive continuance, for two or three days, of the inefficient contraction, marked by frequent pulse, coated tongue and mental wanderings, the Doctor had been forced to relieve his patient by a resort to instruments. When called upon, the labor had commenced; the os uteri was partially dilated, *and not at all rigid*, but the contractions evidently involved different planes of the uterine muscular tissue—first in one part, then in another. From his former experience, the Doctor anticipated trouble and delay. The writer suggested the use of gelseminum, believing that the irregular uterine contractions were due to a want of tone in the sympathetic nervous system. Whether true or not, the result seemingly sustained the theory. Eight drops of the fluid extract were administered at an interval of two hours. After the second dose the uterine contraction became more general, and improvement gradually followed until after the eighth dose, when the patient was delivered, by the unaided forces of nature, of a large, healthy child.—*Trans. Med. Society of Virginia in Virginia Medical Monthly.*

